# Heteroleptic Ir(III)-based near-infrared organic light-emitting diodes with high radiance capacity

**DOI:** 10.1038/s41598-023-27487-6

**Published:** 2023-01-25

**Authors:** Yongjin Park, Gyeong Seok Lee, Woochan Lee, Seunghyup Yoo, Yun-Hi Kim, Kyung-Cheol Choi

**Affiliations:** 1 School of Electrical Engineering, Korea Advanced Institute of Science and Technology (KAIST), 291 Daehak-Ro, Yuseong-Gu, Daejeon, 34141 Republic of Korea; 2 Department of Chemistry and RNIS, Gyeongsang National University, Jinju, 660-701 Republic of Korea

**Keywords:** Engineering, Materials science, Optics and photonics

## Abstract

Near-infrared organic light-emitting diodes (NIR OLEDs) with heavy metals are regularly reported due to the advantages of their various applications in healthcare services, veil authentication, and night vision displays. For commercial applications, it is necessary to look at radiance capacity (RC) instead of radiance because of power consumption. However, recent papers still reported only simple high radiance performance and do not look at device from the point of view of RC. To overcome this hurdle, we designed Ir(III)-based heteroleptic NIR materials with two types of auxiliary ligand. The proposed emitters achieve a highly oriented horizontal dipole ratio (Ir(mCPDTiq)_2_tmd, complex **1**: 80%, Ir(mCPDTiq)_2_acac, complex **2**: 81%) with a short radiative lifetime (**1**: 386 ns, **2**: 323 ns). The device also shows an extremely low turn-on voltage (V_on_) of 2.2 V and a high RC of 720 mW/sr/m^2^/V. The results on the V_on_ and RC of the device is demonstrated an outstanding performance among the Ir(III)-based NIR OLEDs with a similar emission peak.

## Introduction

OLEDs have been successfully established and are widely used as an important light source in conventional display and lighting markets^1^. In particular, OLEDs are pioneering a new market beyond the visible spectral range. Recently, NIR OLEDs are of great interest not only in the field of wearable healthcare systems such as photobiomodulation (PBM)^2–6^ and photodynamic therapies (PDT)^7^ but also for night-vision displays and optical signal processing. In particular, phototherapy studies using OLED have been steadily published in the past decade. It is known that the effect of phototherapy is maximized in the red or NIR wavelength, where the absorption of cytochrome c oxidase (CCO), a photoreceptor, occurs well^8^. Recently, it has been reported that there is a wound healing effect not only in the red region, but also by using NIR OLED^2^. Moreover, a low level light therapy (LLLT) technique, which heals using lower energy than that of PDT, is used in PBM such as wound healing. As a result, the device was driven in a low voltage area (6 V or less) for phototherapy, and a lower voltage is required through integration, such as a battery, to develop actual commercial products. Therefore, it is more important than anything else to realize NIR OLEDs from an RC point of view. Although new NIR emitters are continuously being designed for implementation of NIR OLEDs, the performance of NIR OLEDs is still far behind devices in the visible range due to energy gap law^9,10^. Many studies have been done to overcome the bottleneck of the intrinsic performance of NIR OLEDs, such as using thermally activated delayed fluorescence (TADF)^11–15^ or using transition heavy metals such as Pt(II)^16–20^, Os(II)^21–23^, and Ir(III)^24–30^ to obtain high efficiency. The method using TADF can transfer the non-radiative triplet exciton to the singlet space and obtain 100% of internal quantum efficiency (IQE). Moreover, Pt(II) and Os(II) complexes are extremely expensive to use in comparison to the Ir(III) complex. Conventionally, many transition metal-based NIR OLEDs have been simply reported in terms of high radiance^16^. However, the voltage at maximum radiance is so high that there are many restrictions on applying it to actual industries from the perspective of power consumption. Recently, it has been reported that a cyclopentadithiophene (CPDT) unit has many advantages, such as efficient charge transfer, high electron density and strong electron donor ability due to a structure with high planarity in which bithiophene is fused^31–33^. Therefore, it has been adopted as a unit in photoactive layers and hole transport layers of electronic materials such as organic solar cells and quantum-dot solar cells due to its optimal properties^31–35^.

**Figure 1:**
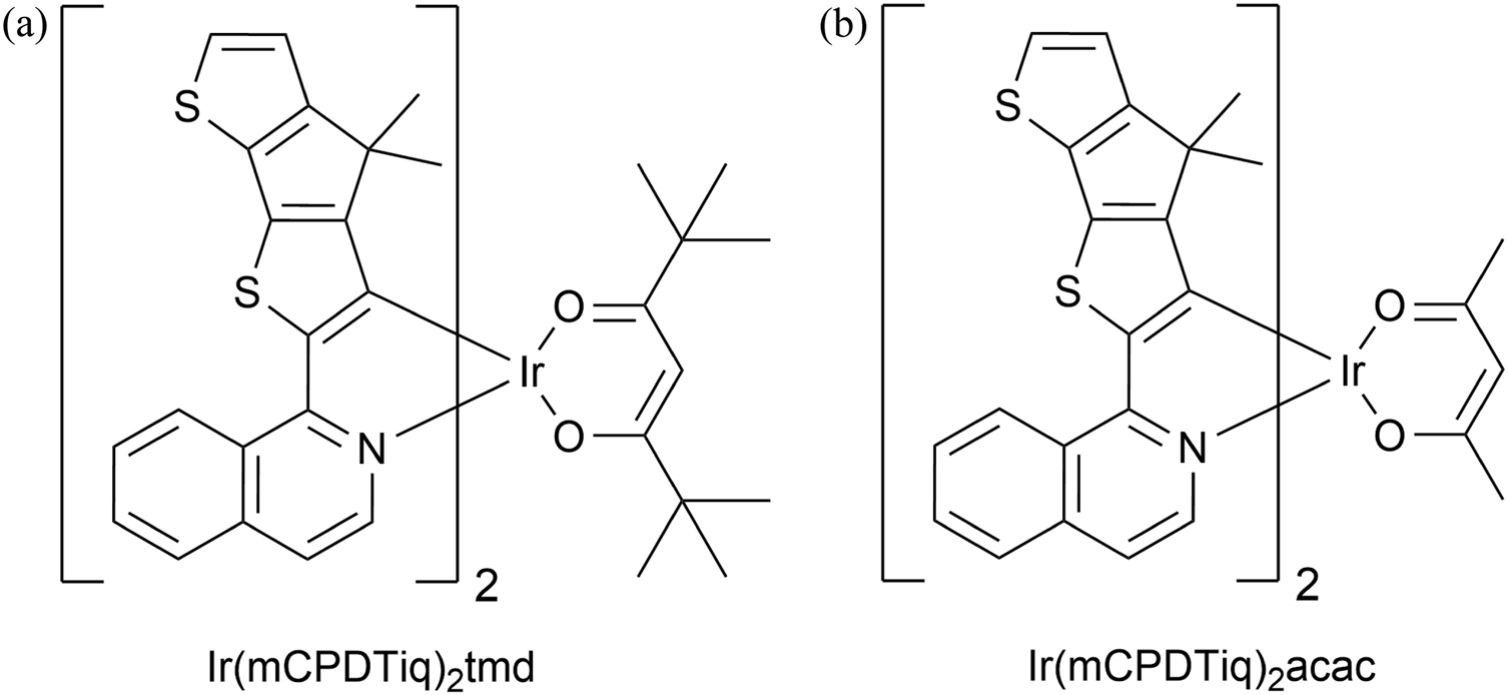
Chemical structures of (**a**) Ir(mCPDTiq)_2_tmd and (**b**) Ir(mCPDTiq)_2_acac.

In this study, CPDT-isoquinoline based Ir(III) complexes for NIR OLED application were synthesized and designed because the CPDT-based ligand has an electron-rich and significant extension of the conjugative structure that enables the emission peak to have a longer wavelength. Therefore, the Ir(III) complex with a CPDT-isoqinoline ligand has a small energy band gap (E_g,_ ≤ 2.1 eV) that is suitable for NIR emission. Furthermore, the substitution of the two methyl groups in sp^3^ carbon of CPDT can suppress the excessive aggregation between molecules^34,36,37^. In addition, we introduced two β-diketone ancillary ligands with long conjugation lengths to increase solubility and facilitate accurate wavelength control, as well as attempt to enhance the horizontal orientation^24,38,39^. Based on the above design concepts, the Ir(mCPDTiq)_2_tmd (complex **1**), Ir(mCPDTiq)_2_acac (complex **2**) were newly developed and chemical structures are shown in Fig. 1. The two compounds achieved maximum emission peaks of 760 nm and 755 nm in dichloromethane solution, respectively.

## Results and discussion

### Synthesis and characterization

**Figure 2:**
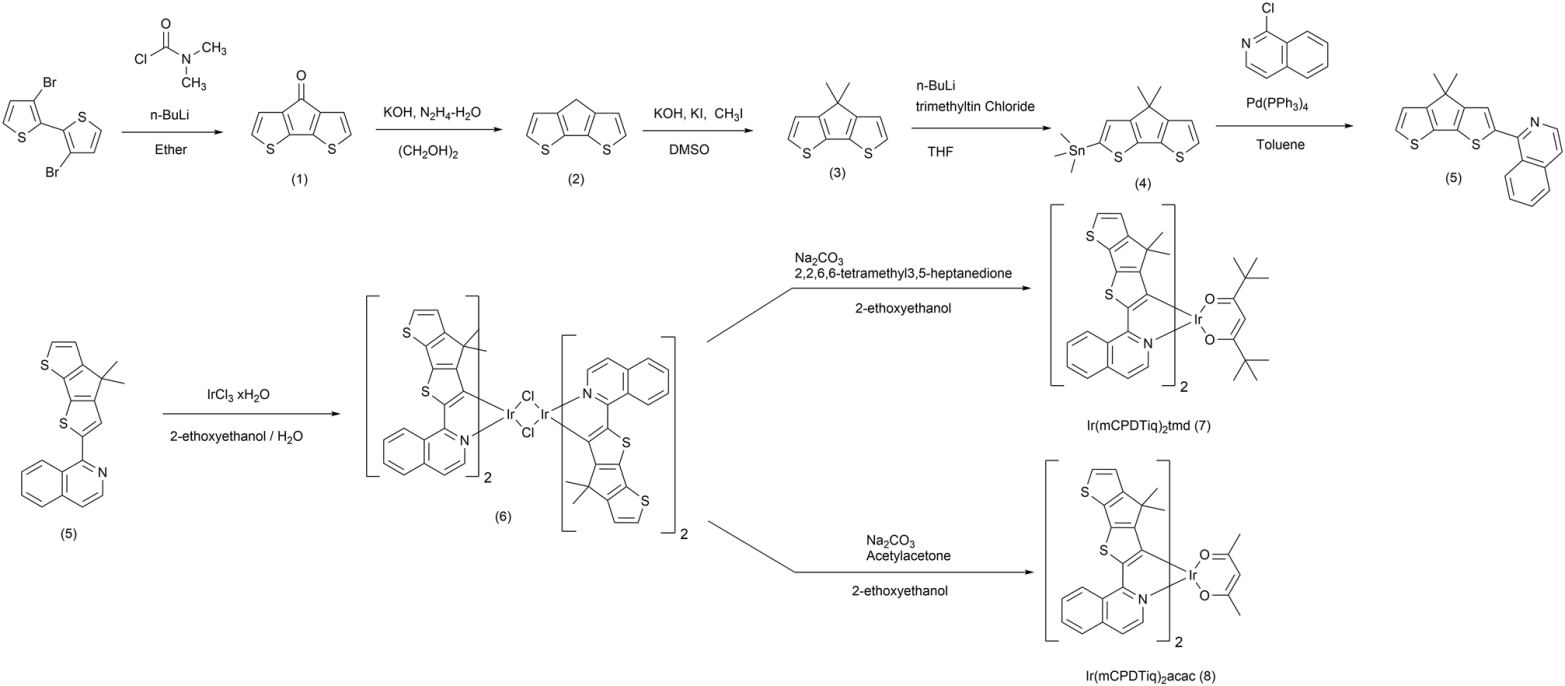
Synthetic scheme of cyclometalating ligand and iridium complexes.

The newly developed iridium complexes were prepared using universal Stille cross-coupling reactions between (4,4-dimethyl-4H-cyclopenta[2,1-b:3,4-b']dithiophen-2-yl)trimethylstannane (4) and 1-chloroisoquinoline. They were formed into two new iridium complexes using different ancillary ligands, as shown in Fig. 2. The bridged dimer was synthesized by a common Nonoyama reaction and a base-catalyzed ligand exchange reaction method using iridium hydrate (IrCl_3_ nH_2_O) and a freshly prepared ligand(1-(4,4-dimethyl-4H-cyclopenta[2,1-b:3,4-b']dithiophen-2-yl)isoquinoline (5)). Then, complex **1** and complex **2** were synthesized from an iridium dimer [(mCPDTiq)_2_Ir(μ-Cl)]_2_ and 2,2,6,6-tetramethyl-3,5-heptanedione (tmd), acetylacetone (acac), respectively. The synthesized iridium complexes were characterized via ^1^H-NMR, ^13^C-NMR spectroscopy and high-resolution mass spectroscopy. All intermediates were also confirmed by ^1^H-NMR, ^13^C-NMR spectroscopy and HR mass methods (see in Supporting Information Figs. S1–S13). We also estimated the thermal properties of the ligand-based iridium complexes using TGA and DSC thermograms (see in Supporting Information Figs. S14–S17). Both complexes (**1** and **2**) showed stoutness up to 340 °C and experienced no phase change up to 250 °C. Overall, the synthesized iridium complexes exhibited sufficient thermal stability.

A cyclic voltammetry (CV) experiment was performed to study the energy level of the newly developed complexes, as shown in Fig. S18. By comparing them with the oxidation potential of the ferrocene, we could determine the highest occupied molecular orbital (HOMO) levels of complexes **1** and **2**. The electrochemical property of these Ir(III) complexes was estimated by CV experiments using tetrabutylammonium perchlorate as an electrolyte in dimethylformaide solution. The HOMO energy level was determined by comparing the oxidation potential of ferrocene, a standard material, and the synthesized materials. Both compounds **1** and **2** showed clear oxidative fluctuation at 0.78, and 0.76 V, respectively. According to the equation in Fig. S18, the HOMO energy levels were confirmed to be − 5.23, and − 5.21 eV, respectively^40^. The band gap energy (E_g_) of both compounds was same value at 2.08 eV, which was obtained from the onset value of the absorption spectra in the solution of each compounds. The LUMO energy levels were calculated by subtracting the band gap energy from the HOMO energy.

### Photophysical properties

**Figure 3:**
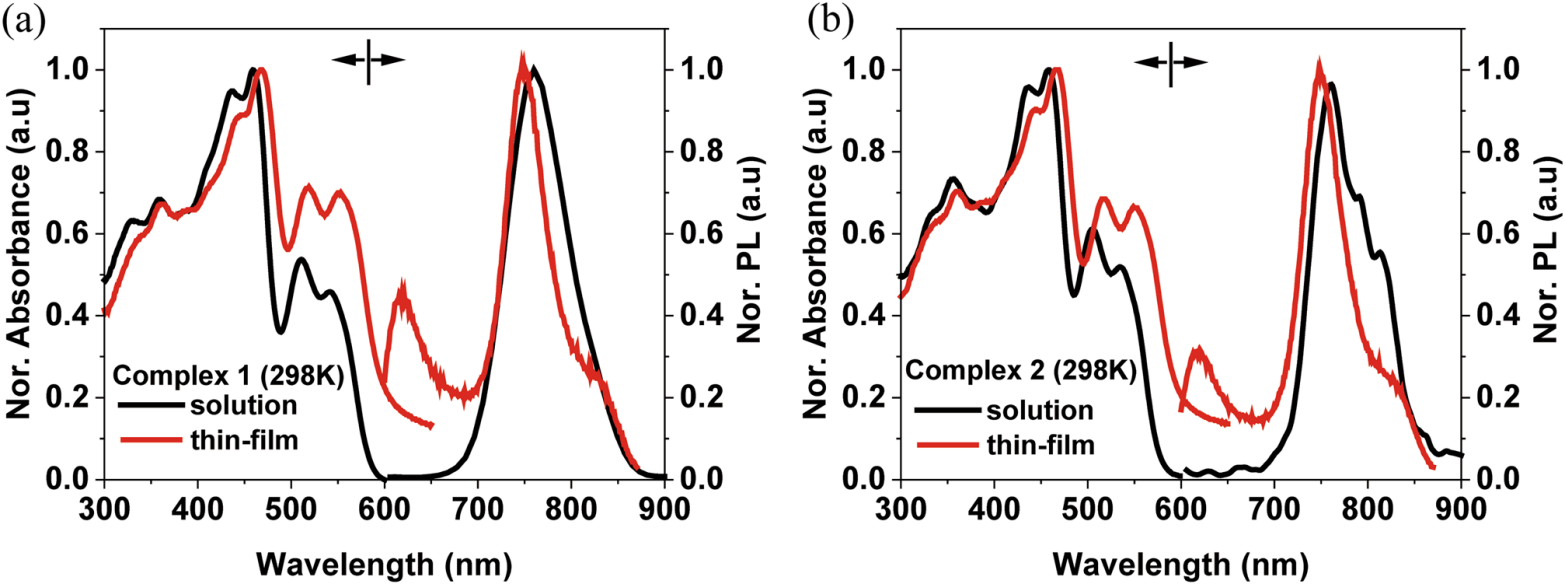
UV–Visible absorbance and PL in solution (black line), thin-film (red line) at room temperature (298 K): (**a**) Ir(mCPDTiq)_2_tmd and (**b**) Ir(mCPDTiq)_2_acac.

Figure 3 show the UV–Visible absorption and photoluminescence spectra of our two complexes **1** and **2** in dichloromethane solution and vacuum-deposited thin-film with the host material at room-temperature (298 K). Spin allowed π–π* transitions were shown to be responsible for the high absorption band below 450 nm. Relatively weak absorption bands in the range 500–700 nm in both spectra were assigned to mixed ^1^MLCT and ^3^MLCT (singlet and triplet metal-to-ligand charge transfer) with an inter-ligand charge transfer (^1^LLCT). The photoluminescence (PL) emission peaks of complexes **1** and **2** in dichloromethane solution were observed at 760 nm with FWHM (full-width at half maximum) of 79 nm and at 755 nm with FHWM of 86 nm, respectively. Complex **1** was slightly 5 nm red shifted compared with complex **2**, which was due to the ancillary ligand effect. The result can be compared with the well-known already reported two iridium complexes Ir(piq)_2_acac (λ_max_ = 622 nm) and Ir(piq)_2_tmd (λ_max_ = 628 nm) based on the phenyl-isoquinoline ligand, and this significant red-shift was due to the electron-rich and significant extension of the conjugative structure of the CPDT unit^41,42^. These Ir(III) complexes show the absolute PL quantum yield (PLQY) and transient decay of complexes **1** (3.2%, 386 ns) and **2** (3%, 323 ns), respectively (see in Fig. S19 for the observed lifetime). Based on the equation below, the radiative and non-radiative constant were calculated.

1 $${k}_{r}=\frac{{\phi }_{p}}{{\tau }_{rad}}, {k}_{nr}=(1-{\phi }_{p})/{\tau }_{rad}$$

All numerical data and PLQY of previous literature are summarized in Table 1 and Table S1, respectively. Although PLQY is slightly low (Table S1), it is noteworthy that the radiative constant of the complexes was significantly lower or similar to those reported in other previous literature^24,26,27^.

**Table 1:** Photophysical properties of Ir(mCPDTiq)_2_tmd and Ir(mCPDTiq)_2_acac.

NIR dopant	$\uplambda$_abs_ (nm) (CH_2_Cl_2_/thin-film)	PL (nm) (CH_2_Cl_2_/thin-film)	${\phi }_{p}$ ^a^	${\tau }_{rad}$ ^b^ ($\mathrm{\mu s}$)	${k}_{r}$ ^c^ (10^5^/s)	${k}_{nr}$ ^d^ (10^5^/s)	HOMO^e^/LUMO^f^ (eV)
Ir(mCPDTiq)_2_tmd	510/430	760/760	0.032	0.39	0.82	24.8	− 5.23/− 3.15
Ir(mCPDTiq)_2_acac	530/430	755/760	0.030	0.32	0.94	30.3	− 5.21/− 3.13

^a^PLQY was measured in vacuum-deposited thin-film (50 nm).

^b^Radiative decay rate measured in dichloromethane (DCM) .

^c^$${k}_{r}={\phi }_{p}/{\tau }_{rad}$$.

^d^$${k}_{nr}=(1-{\phi }_{p})/{\tau }_{rad}$$.

^e^HOMO was recorded using electrochemical CV method.

^f^LUMO was obtained through E_g_ and HOMO level.

**Figure 4:**
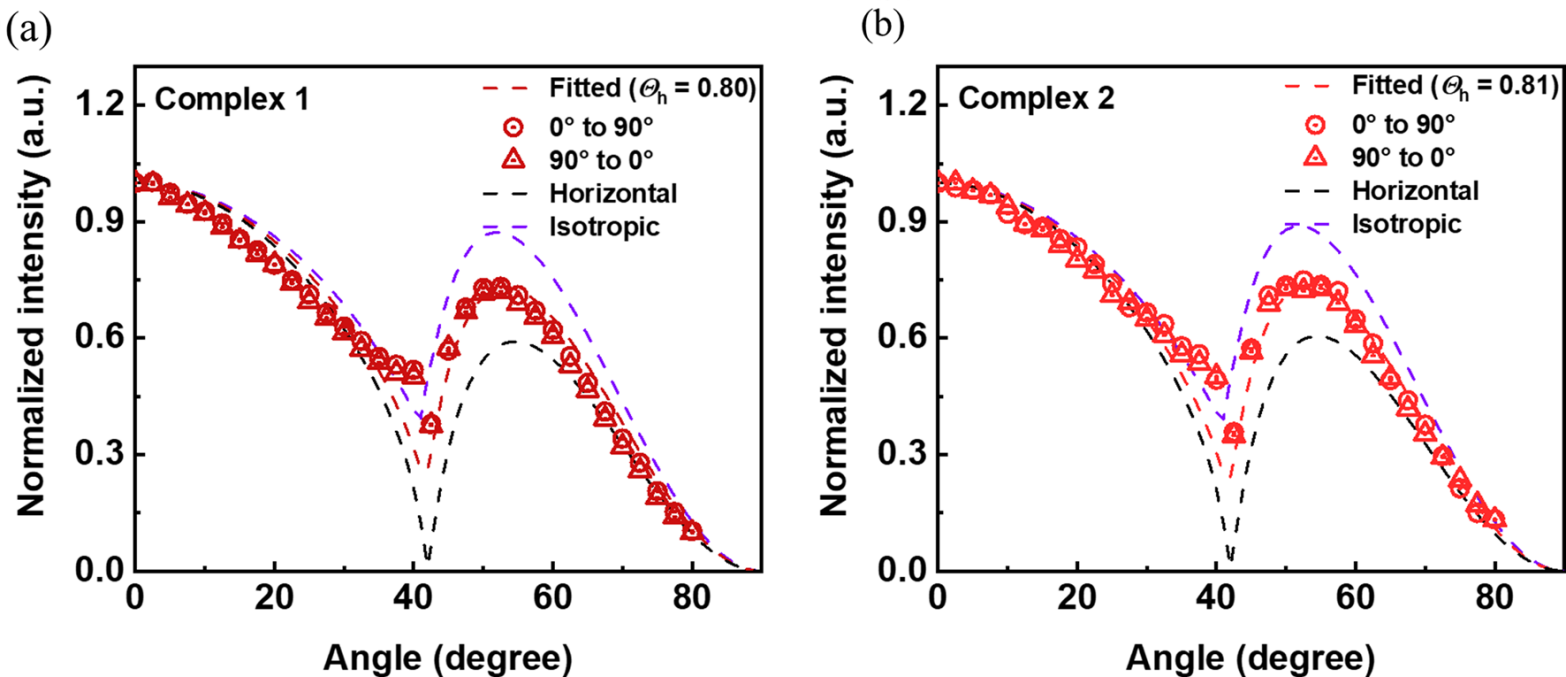
Angular dependence of PL intensity of (**a**) Ir(mCPDTiq)_2_tmd, (**b**) Ir(mCPDTiq)_2_acac. Fitted line (experimental data), horizontal and isotropic line (simulation) of dipole orientation.

To determine how the organic molecules are oriented when deposited as a thin film, angle dependent *p*-polarized photoluminescence spectrum with the given emitters deposited on the substrate was measured, as shown in Fig. 4. Birefringence of each complex was considered for the theoretical fitting. The horizontal dipole orientation (Θ_h_) of complexes **1** and **2** were 0.80 and 0.81, which were highly horizontally oriented compared to the other organic emitters and well matched to the fitted curve, respectively. Complexes **1** and **2** were preferred to be oriented horizontally because they exhibited a heteroleptic structure. The structure of the auxiliary ligand affects the dipole moment of the emitters to be arranged horizontally, thereby enhancing the device characteristics^43,44^.

### Electroluminescence (EL) performance (device performances)

The short radiative lifetimes, efficient NIR emissions and highly horizontal oriented Ir(III) complexes prompted us to look into their EL characteristics further. The device structure of the OLEDs was as follows: indium tin oxide (ITO)/molybdenum trioxide (MoO_3_, 10 nm)/N,N′-Di(1-naphthyl)-N,N′-diphenyl-(1,1′-biphenyl)-4,4′-diamine (NPB, 40 nm)/Bebq_2_: NIR dopant (**x** wt%, 30 nm)/bathophenanthroline (Bphen, 20 nm)/Liq (1 nm)/Al (100 nm). ITO was patterned on a glass substrate. In the device structure, MoO_3_, NPB, Bebq_2_, Bphen, Liq, and Al were used for the hole injection layer (HIL), hole transport layer (HTL), host material, electron transport layer (ETL), electron injection layer (EIL), and cathode, respectively. In particular, Bebq_2_ is well known to act as an effective host during the doping process with the iridium complex and to facilitate energy transfer^45,46^.

**Figure 5:**
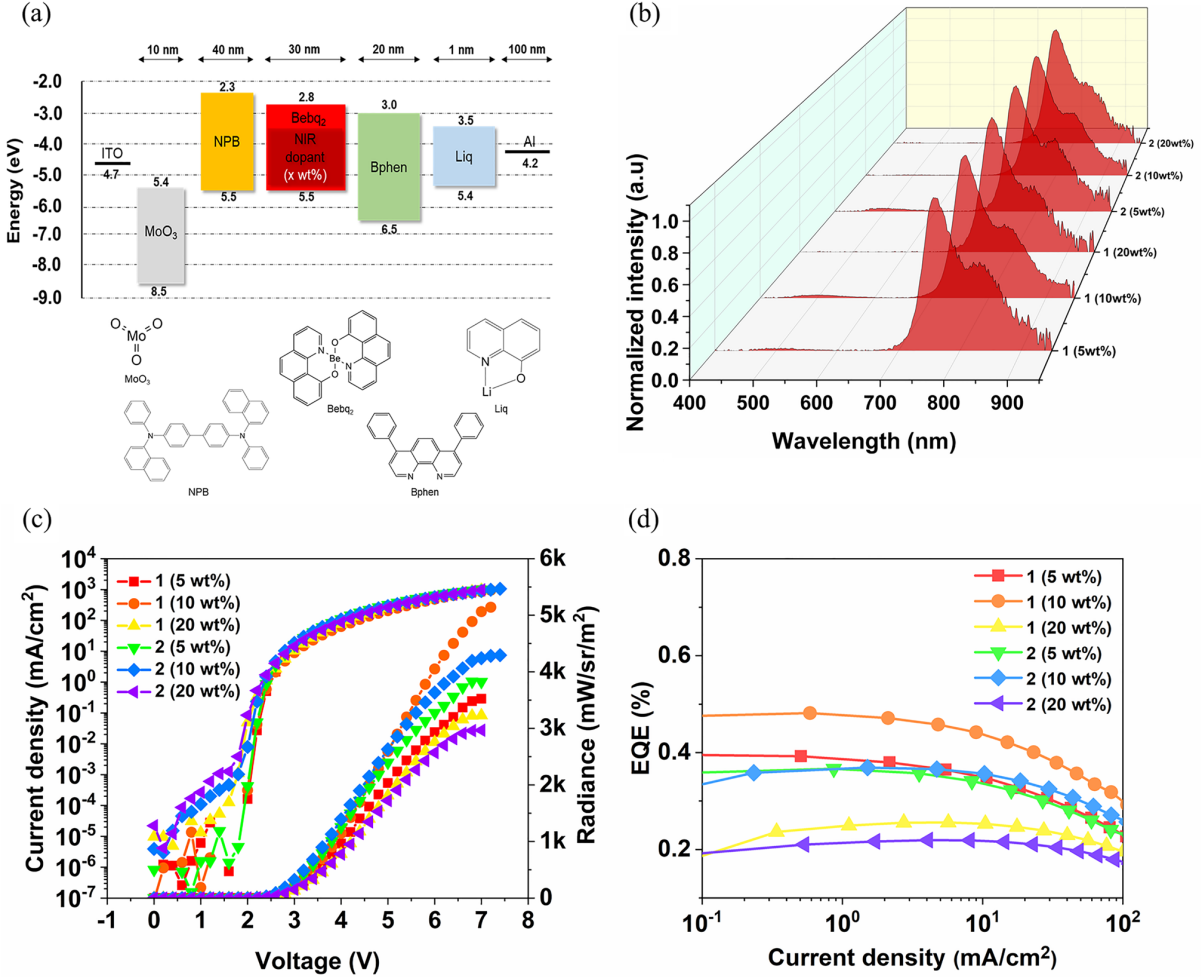
Electrical and optical properties of the NIR OLEDs. (**a**) Energy level alignment diagram and molecular structure (**b**) EL spectra by concentration (**c**) J–V–R (**d**) EQE of the NIR OLEDs depending on the doping concentrations.

The device optimization procedure was carried out with different doping concentrations (x = 5, 10, and 20 wt%) of the NIR dopant (complexes **1**, **2**). Moreover, all the devices were encapsulated with Al_2_O_3_ (30 nm) using the atomic layer deposition (ALD) method to measure the lifetime. The energy level and molecular structure of the materials and schematic device structure are shown in Fig. 5a. The materials of each layer were selected in consideration of the energy level, and the structure was designed to facilitate charge balance and lower driving voltage.

The normalized EL spectrum of the NIR OLEDs are shown in Fig. 5b as a function of the complexes with the doping concentration. The EL spectra of all the devices are identical to PL, suggesting that exciton was well confined inside the EML. However, the phenomenon in which a peak appears in a visible region around 500 nm was observed according to the doping concentration. The peak in the visible area decreases when more dopant is injected at a high concentration. This phenomenon could be explained by the fact that the host material’s (Bebq_2_) PL peak (see Fig. S20) emerges at low doping concentrations. The higher the concentration, the easier it is for energy to transfer from the host to the dopant, thus the peak in the visible light area decreases^47^ (see Fig. 5b, Fig. S21).

The current density–voltage–radiance (J–V–R), light distribution characteristics and EQE are shown in Fig. 5c,d. All devices ran neatly without leakage current and had a Lambertian emission distribution (Fig. S22). All numerical data are described in Table 2. The maximum RC was 723 and 606 mW/sr/m^2^/V, EQE_max_ was 0.48 and 0.37%, and V_on_ was extremely low at 2.2 V for complexes **1** and **2** at a doping concentration of 10 wt%, respectively. In addition, the stability and reliability after over 120 h based on median Lethal Time (LT50) were proved for complexes **1** and **2** at 10 wt%, respectively (Fig. S23).

**Table 2:** Characteristics of NIR OLEDs according to ligand and doping ratio.

Dopant	Ratio (wt%)	EQE_max_ (%)	V_on_ (V)	R_max_ (mW/sr/m^2^)	V_max_ (V)	RC (mW/sr/m^2^/V)
Ir(mCPDTiq)_2_tmd	5	0.40	2.4	3520	7	503
	10	0.48	2.2	5060	7	723
	15	0.26	2.2	3230	7	461
Ir(mCPDTiq)_2_acac	5	0.37	2.4	3820	7	546
	10	0.37	2.2	4240	7	606
	15	0.22	2.2	2970	7	424

Both complexes showed the highest performance at 10 wt%, indicating that they run at significantly lower voltages compared to the previously reported NIR OLEDs, which means that a balanced charge was supplied and transported to the device as an optimal structure, resulting in direct trapping of the carrier.

The NIR OLED developed through in study showed the best performance for complex **1** at a doping concentration of 10 wt%. The numerical values (R_max_, V_max,_ and RC) of previous studies are presented in Table S2. Among them, the RC was calculated by dividing the maximum radiance by the maximum voltage. Compared to the other reported Ir(III)-based NIR OLED in the literature, there are some deficiencies in the highest radiance and EQE due to low the PLQY, but there is the advantage of a high RC. This is presumed to be caused by the fact that the current density of the NIR OLED device developed in this study is about 20 times high. Because the EQE is affected by the PLQY, it may be low when the PLQY is low. However, through optimization of the overall structure of the device, it was possible to implement a device with a high RC by achieving a low driving voltage and a high radiance at a relatively low voltage due to good current injection.

**Figure 6:**
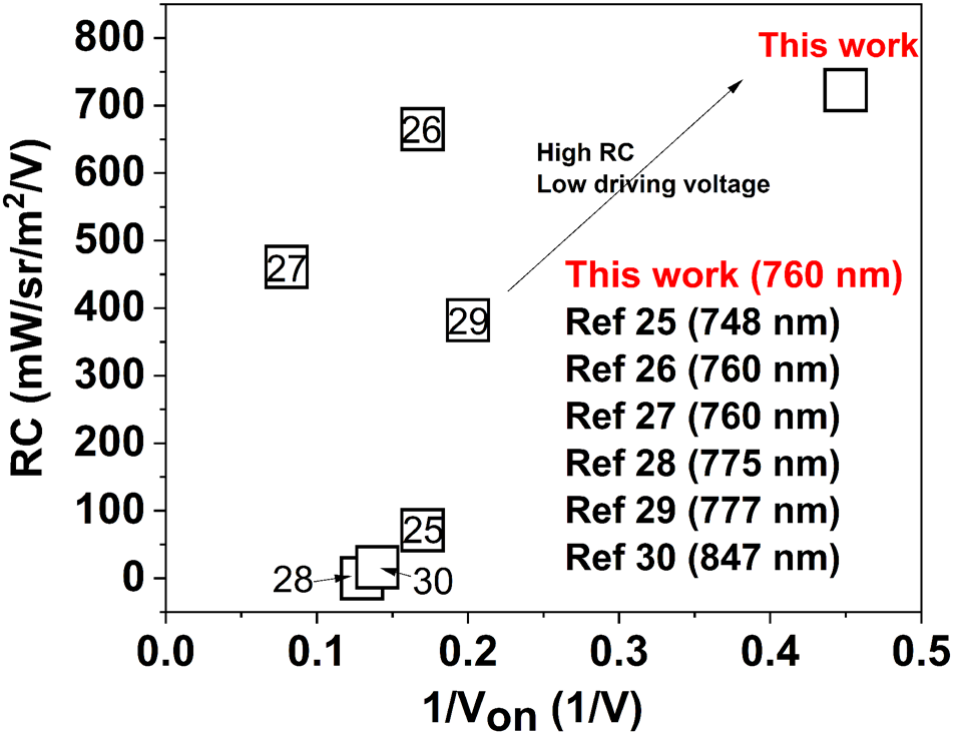
Radiance capacity depending on V_on_ (Plotted by referring to NIR OLEDs based on Ir(III) complex).

Basically, a high radiance is not very important in phototherapy using LLLT. Rather, the device must be manufactured in terms of the RC taking into consideration the driving voltage for the corresponding radiance for commercial applications. In other words, it is tremendously important to consider the power consumption of NIR OLEDs for them to be used in actual applications. The NIR OLED had an extremely low V_on_ and the highest RC compared to the other Ir(III)-based NIR OLEDs shown in Fig. 6.

## Conclusion

In summary, heteroleptic Ir(III) complexes are designed and synthesized with a high horizontal dipole ratio of dipole orientation (complex **1**: 80%, complex **2**: 81%) and a short radiative lifetime (complex **1**: 390 ns, complex **2**: 320 ns). Because of its high electron density and long π conjugation length, this structure can close the energy gap and emit the NIR wavelength (760 nm). Notably, the NIR OLED with our newly designed Ir(III) complex has a significantly lower V_on_, overwhelmingly high RC, and better reliability than previous Ir(III) complex-based NIR OLEDs. Hence, our study serves to present the future direction of NIR OLEDs for practical applications in the industry.

## Methods

### General information and materials

All starting reactants were purchased from Sigma Aldrich and Alfa Aesar. The palladium catalyst (Tetrakis(triphenylphosphine)palladium(0)) was purchased from Umicore. Iridium hydrate was supplied by Furuya Metal Korea. All commercially purchased reagents were used without any further purification, and a series of intermediates were synthesized according to the reported method^48^. Moreover, (4,4-dimethyl-4H-cyclopenta[2,1-b:3,4-b']dithiophen-2-yl)trimethylstannane (4) was prepared according to the literature^49^.

### Measurement

All spectra (^1^H NMR and ^13^C NMR) were measured using a 300 MHz (Bruker) and a 500 MHz (DRX) spectrometer. Mass spectra (FAB and EI data) were acquired with a high-resolution mass spectrometer using a JMS-700 (Jeol). To analyze the thermal stability of prepared iridium complexes, thermogravimetric analysis was conducted using TA 2050 TGA instruments. All differential scanning calorimetry (DSC) data were measured using a TA Instruments 2100 DSC and heated from room temperature to 800 °C (10 °C/min.) Ultraviolet–visible absorption spectra were acquired on a LAMBDA-900 spectrophotometer (PerkinElmer). Cyclic voltammetry (CV) was measured to study the electrochemical properties of the prepared materials. A three-electrode system was used with a carbon glass electrode, an Ag/AgCl electrode and platinum wire as the working electrode, reference electrode and counter electrode, respectively. For detailed experimental conditions, a cyclic voltammogram was recorded using tetrabutylammonium perchlorate as an electrolyte in a 0.1 M dimethylformamide solution under nitrogen gas at a scan rate of 50 mV/s.

### Synthesis of 4H-cyclopenta[2,1-b:3,4-b’] dithiophen-4-one (1)

This reaction was done following the literature method^48^. ^1^H NMR (300 MHz, CDCl_3_): δ (ppm) = 7.07 (d, *J* = 4.8 Hz, 2 H), 7.02 (d, *J* = 4.8 Hz, 2 H).

### Synthesis of 4H-cyclopenta[2,1-b:3,4-b’] dithiophene (2)

This reaction was done following the literature method^48^. ^1^H NMR (300 MHz, CDCl_3_): δ (ppm) = 7.21 (d, *J* = 5.1 Hz, 2 H), 7.12 (d, *J* = 5.1 Hz, 2 H), 3.5 (s, 2 H).

### Synthesis of 4,4-dimethyl-4H-cyclopenta[2,1-b:3,4-b’] dithiophene (3)

This reaction was done following the literature method^48^. ^1^H NMR (300 MHz, CDCl_3_): δ (ppm) = 7.19 (d, *J* = 4.8 Hz, 2 H), 7.02 (d, *J* = 4.8 Hz, 2 H), 1.48 (s, 6 H).

### Synthesis of (4,4-dimethyl-4H-cyclopenta[2,1-b:3,4-b’] dithiophen-2-yl) trimethylstannane (4)

This reaction was done following the literature method^49^. ^1^H NMR (300 MHz, CD_2_Cl_2_): δ (ppm) = 6.98 (d, *J* = 4.8 Hz, 1 H), 6.90 (s, 1 H), 6.84 (d, *J* = 4.8 Hz, 1 H), 1.27 (s, 6 H), 0.31—0.12 (m, 9 H).

### Synthesis of 1-(4,4-dimethyl-4H-cyclopenta[2,1-b:3,4-b']dithiophen-2-yl)isoquinoline (5)

(4,4-dimethyl-4H-cyclopenta[2,1-b:3,4-b']dithiophen-2-yl)trimethylstannane (10.0 g, 48.5 mmol), 1-choloroisoquinoline (7.9 g, 48.5 mmol) were dissolved in toluene (500 mL) and purged with nitrogen gas for 30 min. Then a palladium catalyst (Pd(PPh_3_)_4_, 1.1 g, 1.0 mmol) was added to the reaction mixture and stirred at 105 °C for 12 h. After cooling to room temperature, the reactant mixture was quenched by water and it was extracted with methylene chloride three times. Then the organic solution portion was dried using anhydrous magnesium sulfate and evaporated. After solvent was removed, the product was purified by column chromatography using hexane and ethyl acetate (Yield: 7.4 g, 82%). ^1^H NMR (300 MHz, CD_2_Cl_2_): δ (ppm) = 8.54 (d, *J* = 8.4 Hz, 1 H), 8.40 (d, *J* = 5.7 Hz, 1 H), 7.80 (d, *J* = 7.5 Hz, 1 H), 7.65–7.54 (m, 3 H), 7.47 (d, *J* = 5.4 Hz, 1 H), 7.19 (d, *J* = 4.8 Hz, 1 H), 6.99 (d, *J* = 4.8 Hz, 1 H), 1.46 (s, 6 H). ^13^C NMR (300 MHz, CD_2_Cl_2_): δ (ppm) = 161.42, 161.10, 153.41, 143.63, 142.07, 138.06, 137.24, 135.31, 129.99, 127.64, 127.29, 126.42, 126.35, 125.54, 122.84, 120.95, 119.23, 52.71, 45.38, 24.94 HRMS-EI + (m/z): [M] + calcd for C_20_H_15_NS_2_, 333.06; found, 333.0648.

### Synthesis of Ir(III) dimer [(mCPDTiq)_2_Ir(μ-Cl)]_2_ (6)

A cyclometalating ligand, 1-(4,4-dimethyl-4H-cyclopenta[2,1-b:3,4-b']dithiophen-2-yl)isoquinoline (5) (7.5 g, 22.5 mmol) and iridium(III) chloride hydrate (3.1 g, 10.2 mmol) were dissolved in ethyl glycol and distilled water. The reaction mixture was stirred overnight under nitrogen gas. Then, the precipitated solids were filtered and washed with water and a small amount of diethyl ether. These crude products were thoroughly dried and then used without a further purifying procedure.

### Synthesis of Ir(mCPDTiq)_2_ (tmd) (7)

A prepared μ-chloro-bridged dimer [(mCPDTiq)_2_Ir(μ-Cl)]_2_ (5 g, 2.8 mmol), 2,2,6,6-tetramethyl-3,5-heptanedione (2.1 g, 11.2 mmol) and sodium carbonate (2.9 g, 28.0 mmol) were dissolved in ethyl glycol. The three-neck flask was refluxed for 12 h at 120 °C, and then the cooled reaction mixture was poured into iced water and the precipitated solid was filtered and the filtered solids were extracted with dichloromethane and water. The organic portion was dried using anhydrous magnesium sulfate and evaporate under reduced pressure. After removal of the organic solvent, the black-purple product was purified by column chromatography using hexane and ethyl acetate (Yield: 1.2 g, 42%). ^1^H NMR (300 MHz, CD_2_Cl_2_, d_2_): δ (ppm) = 9.01–8.98 (m, 2 H), 7.97 (d, *J* = 6.6 Hz, 2 H), 7.87–7.83 (m, 2 H), 7.75–7.71 (m, 4 H), 7.16–7.14 (d, *J* = 4.8 Hz, 2 H), 7.12 (d, *J* = 5.1 Hz, 1 H), 5.51 (s, 1 H), 1.58 (s, 12 H), 0.79 (s, 18 H). ^13^C NMR (500 MHz, CD_2_Cl_2_): δ (ppm) = 194.34, 167.44, 165.19, 164.68, 149.95, 141.86, 141.67, 138.16, 136.92, 133.62, 130.81, 127.19, 126.98, 126.57, 126.43, 124.15, 120.68, 114.50, 93.57, 91.89, 91.57, 89.98, 86.87, 85.78, 85.41, 54.14, 53.9, 53.78, 53.63, 53.42, 53.26, 53.21,53.06, 52.70, 45.78, 40.90, 27.57, 23.99, 22.91. HRMS-FAB + (m/z): [M + H] + calcd for C_51_H_47_IrN_2_O_2_S_4_, 1040.21; found, 1040.2174.

### Synthesis of Ir(mCPDTiq)_2_ (acac) (8)

(Yield: 34%) ^1^H NMR (300 MHz, CD_2_Cl_2_, d_2_): δ (ppm) = 9.02–8.98 (m, 2 H), 8.09 (d, *J* = 7.8 Hz, 2 H), 7.91–7.88 (m, 2 H), 7.78–7.75 (m, 4 H), 7.20–7.18 (d, *J* = 6.6 Hz, 2 H), 7.17–7.16 (d, *J* = 4.8 Hz, 2 H), 6.76–6.74 (d, *J* = 4.8 Hz, 2 H), 5.37 (s, 1 H), 1.77 (s, 6 H), 0.80 (s, 6 H), 0.45 (s, 6H). ^13^C NMR (300 MHz, CDCl_3_): δ (ppm) = 184.68, 167.21, 164.75, 142.96, 141.75, 138.36, 137.02, 133.68, 130.90, 127.21, 127.05, 126.55, 124.24, 120.61, 114.71, 100.59, 45.87, 28.43, 24.03, 23.05. HRMS-FAB + (m/z): [M + H] + calcd for C_45_H_35_IrN_2_O_2_S_4_, 956.1211; found, 956.1216.

### UV–vis absorbance and PL measurements

Thin-films (50 nm) were vacuum deposited on a glass substrate under a high vacuum (2 $\times$ 10^−6^ torr). The thickness of the complexes was measured using a quartz thickness-monitor. Then, UV–vis absorption and PL spectra were measured using a Fluorolog-3 (HORIBA). The excitation wavelength was set to 430 nm in both complexes. Moreover, the PLQY of the Ir(III) complexes was measured using a Fluorolog-3 (HORIBA).

### Measurement of the angle dependent p-polarized photoluminescent spectrum (ADPL)

The emission layer was deposited on a bare 50 nm-thick glass substrate. Then, glass encapsulation was done in a nitrogen (N_2_)-filled glove box to avoid degradation from the air. A full angle dependent *p*-polarized PL spectrum was obtained with the goniometer based motorized intensity measurement system (Phelos, Fluxim). Simulation and fitting with the experimental data were made with a customized MATLAB code describing the coherent dipole radiation theory.

### Device fabrication and measurement

All organic and inorganic materials were purchased from Sigma-Aldrich and iTASCO. The devices were fabricated on a patterned ITO glass substrate with a sheet resistance of 15 $\Omega$ per square. MoO_3_, NPB, Bphen, Liq, and Al were deposited at a rate of 0.5, 1, 1, 0.1, and 2 Å/s, respectively. The host (Bebq_2_) and guest (complexes **1** and **2**) were co-deposited at various doping concentrations under a high vacuum of 2 $\times$ 10^−6^ torr. All the devices were encapsulated within Al_2_O_3_ using the atomic layer deposition (ALD) process under 70℃. The current density–voltage-radiance (J–V–R) and angle-resolved EL intensity characteristics of the fabricated devices were obtained with a source-measure unit (Keithley 2400) using a calibrated photodiode (FDS 100, Thorlab) and a fiber optic spectrometer (BW_UVNb, StellarNet) held on a motorized goniometer (PRM1/MZ8, Thorlabs) in a nitrogen (N_2_)-filled glove box. The EQE (*η*_EQE_) and PE (*η*_PE_) of the devices were estimated from the measured full angular characteristics without Lambertian simplification^50^. The operating lifetime was monitored using a McScience Polaronix M6000 OLED Lifetime Tester.

## Supplementary Information

Supplementary Information.

## Data Availability

The datasets used and/or analyzed during the current study available from the corresponding author on reasonable request.
