# Supplementary material for: Heteroleptic Ir(III)-based near-infrared organic light-emitting diodes with high radiance capacity
Source: Sci Rep. 2023 Jan 25;13:1369. doi: 10.1038/s41598-023-27487-6 (PMC9877021; doi:10.1038/s41598-023-27487-6)
Supplement: Supplementary file 1 — Supplementary Information. [file 41598_2023_27487_MOESM1_ESM.docx]

Supporting Information

**Heteroleptic Ir(III)-based Near-Infrared Organic Light-Emitting Diodes with High Radiance Capacity**

*Yongjin Park^1,3^, Gyeong Seok Lee^2,3^, Woochan Lee^1,3^, Seunghyup Yoo^1^*, Yun-Hi Kim^2^*, and Kyung-Cheol Choi^1^**

^1^ School of Electrical Engineering, Korea Advanced Institute of Science and Technology (KAIST), 291 Daehak-ro, Yuseong-gu, Daejeon 34141, Republic of Korea.

^2^Department of Chemistry and RNIS, Gyeongsang National University, Jinju 660-701, Republic of Korea

^3^ These authors contributed equally: *Yongjin Park, Gyeong Seok Lee, and Woochan Lee*.

^*^ Corresponding author.

E-mail addresses: [syoo.ee@kaist.edu](mailto:syoo.ee@kaist.edu) (S. Yoo), [ykim@gnu.ac.kr](mailto:ykim@gnu.ac.kr) (Y. -H. Kim), [kyungcc@kaist.ac.kr](mailto:kyungcc@kaist.ac.kr) (K. -C. Choi).

**
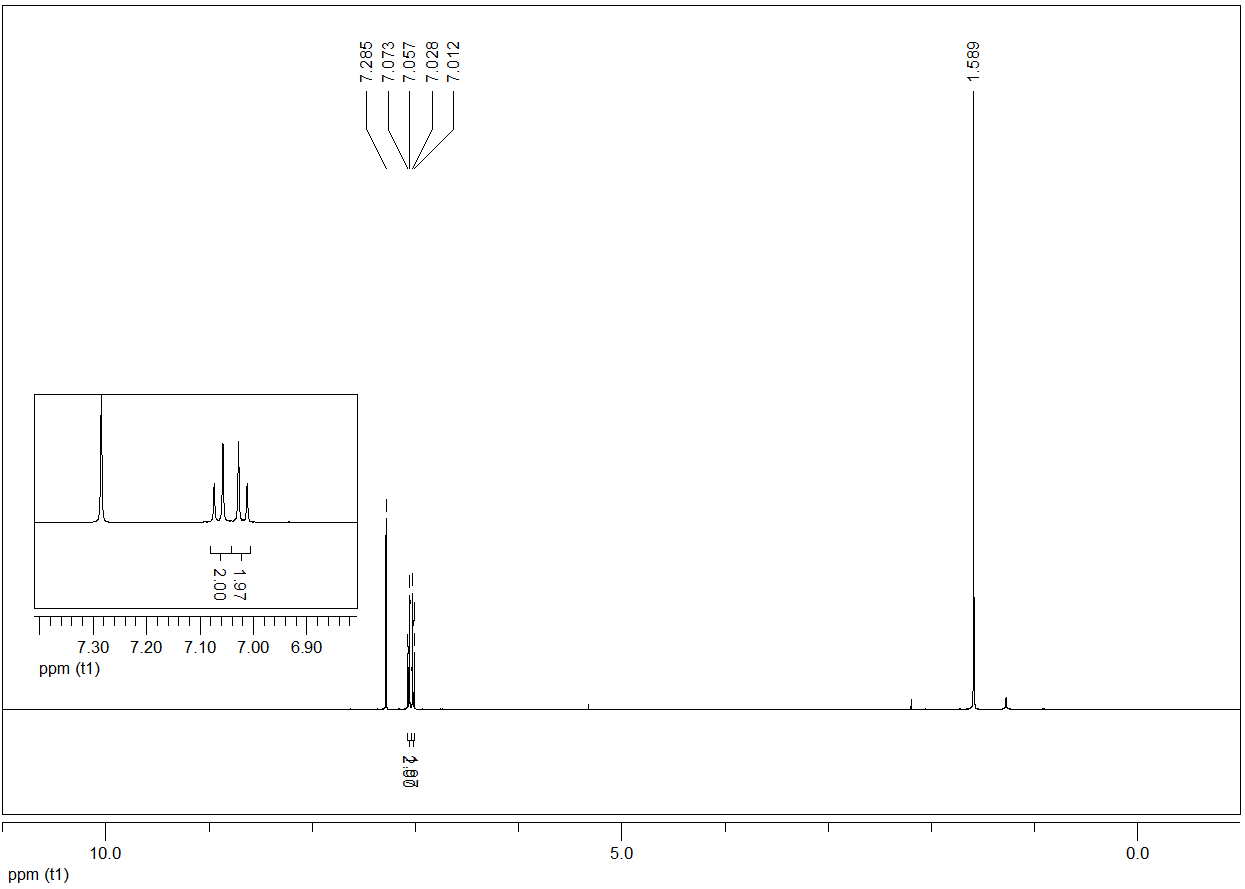
**

**Figure S1.** ^1^H-NMR data of 4H-cyclopenta[2,1-b:3,4-b']dithiophen-4-one (1)


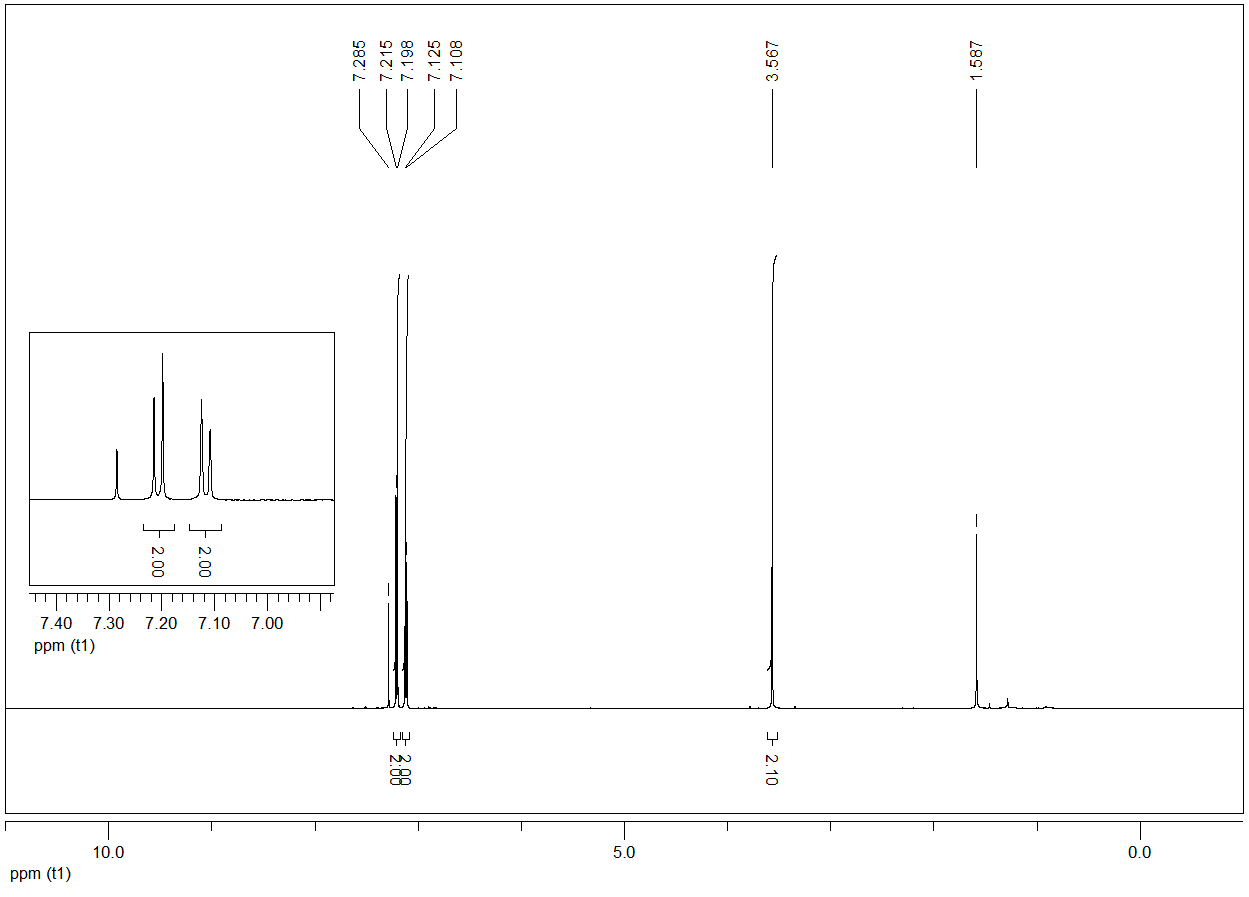


**Figure S2.** ^1^H-NMR data of 4H-cyclopenta[2,1-b:3,4-b']dithiophene (2)


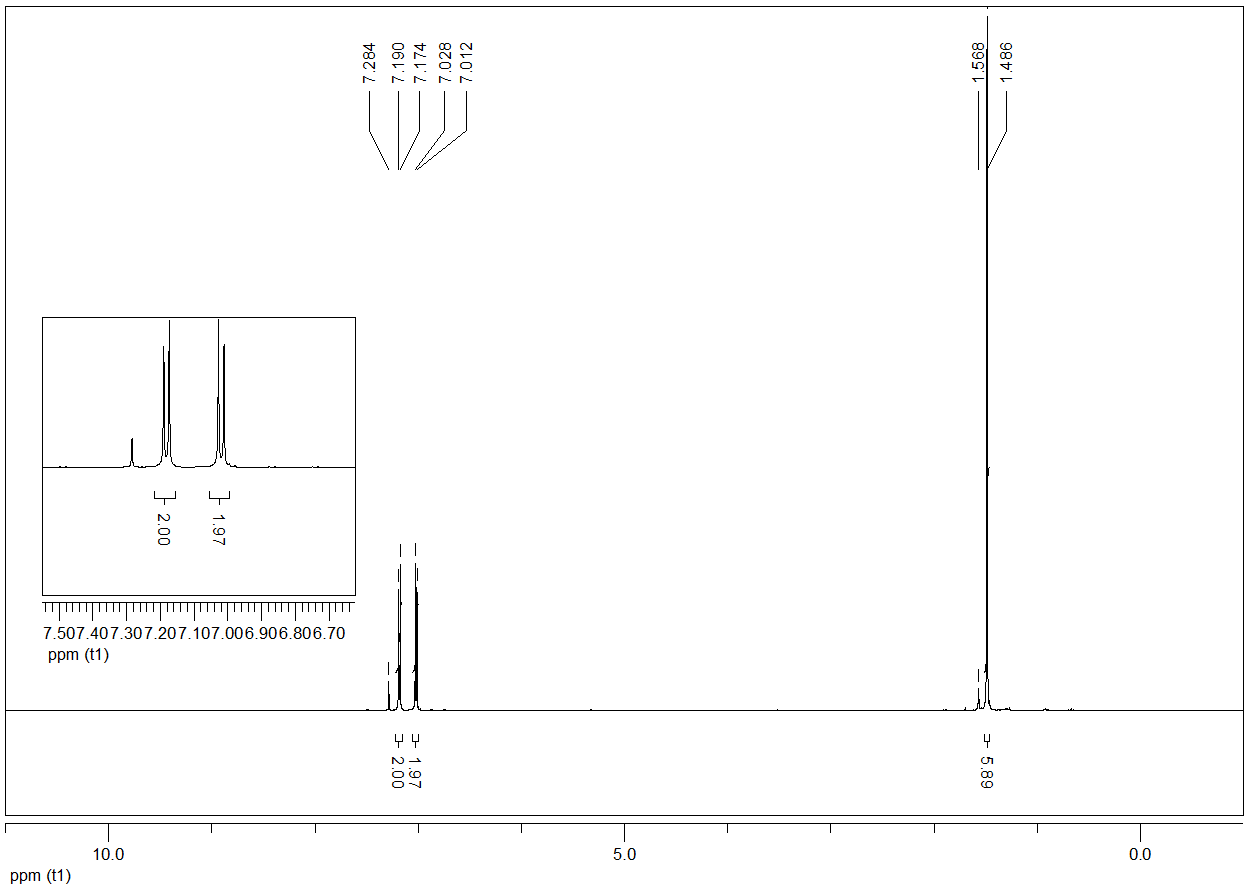


**Figure S3.** ^1^H-NMR data of 4,4-dimethyl-4H-cyclopenta[2,1-b:3,4-b']dithiophene (3)


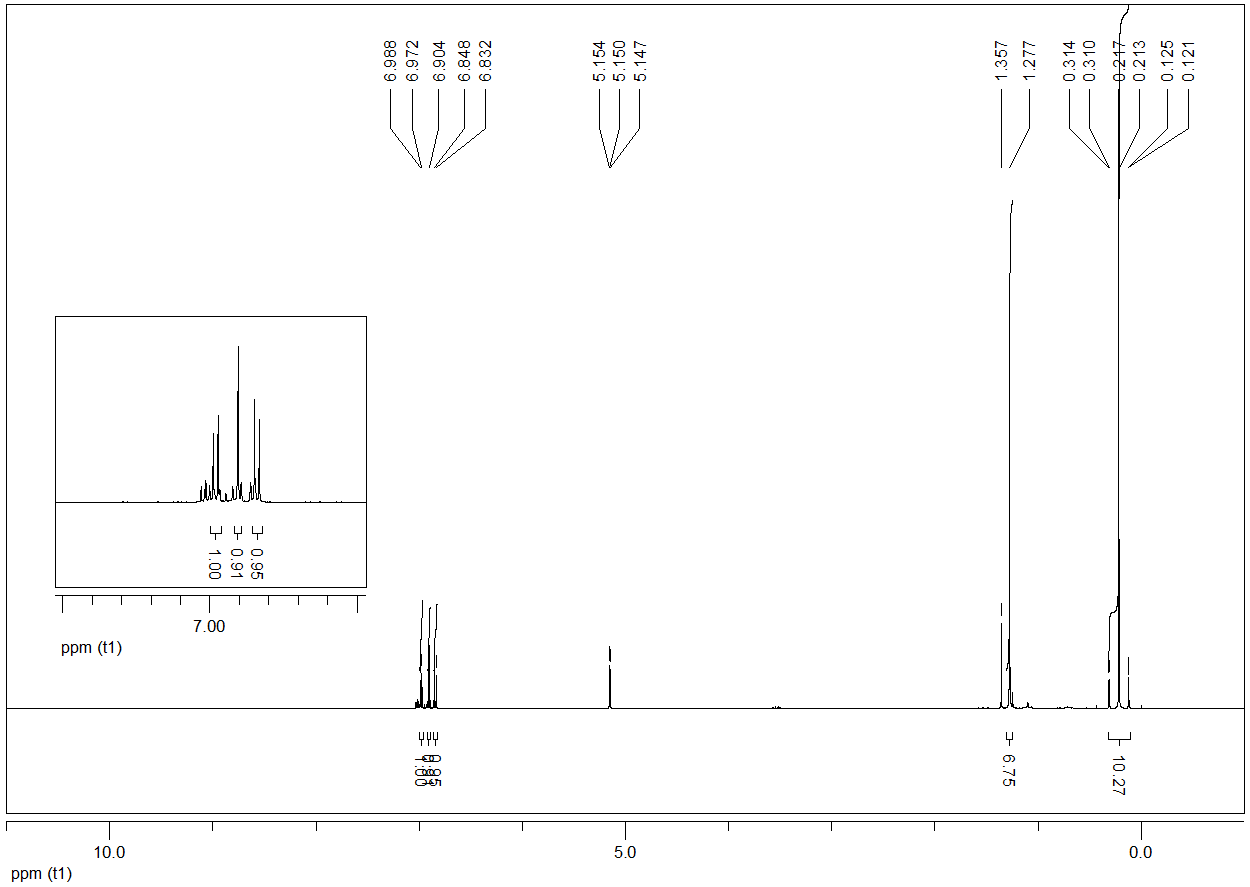


**Figure S4.** ^1^H-NMR data of (4,4-dimethyl-4H-cyclopenta[2,1-b:3,4-b']dithiophen-2-yl)trimethylstannane (4)


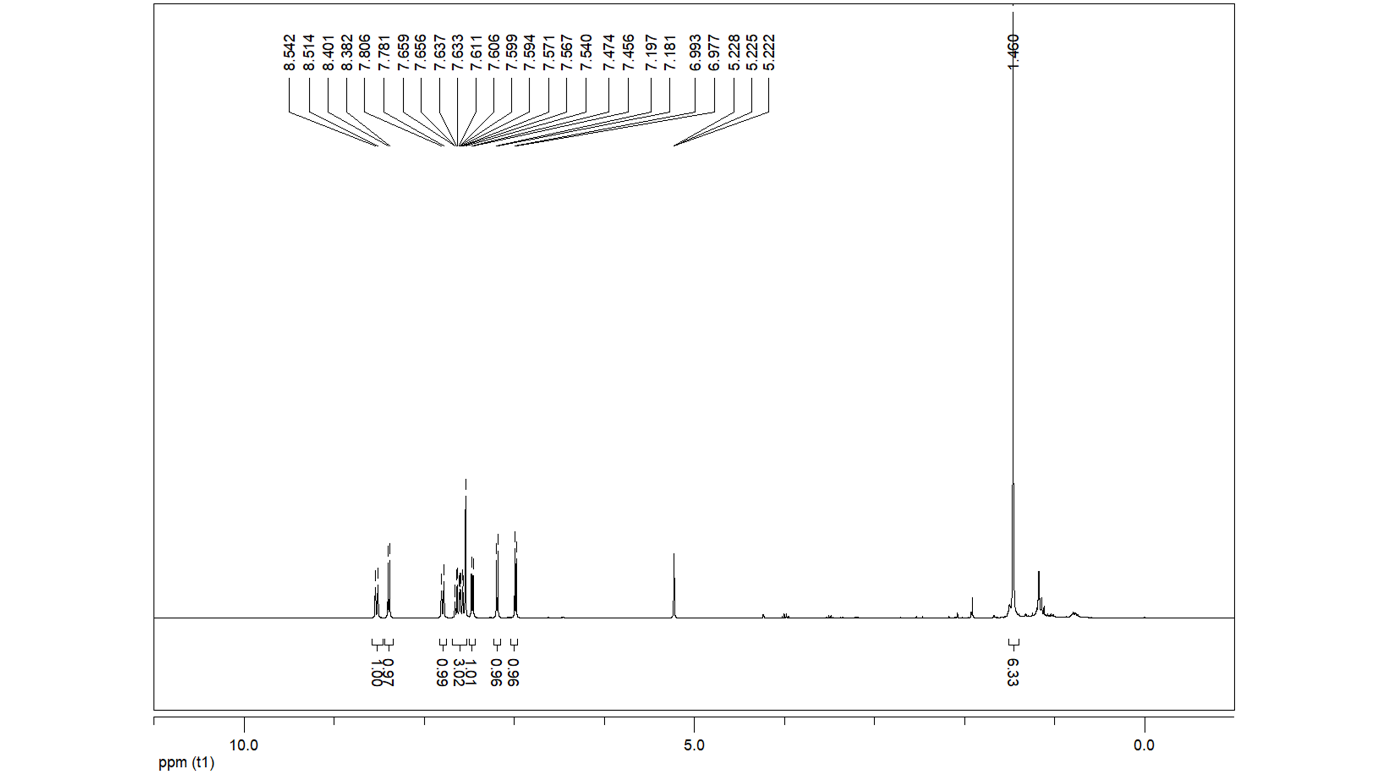


**Figure S5.** ^1^H-NMR data of 1-(4,4-dimethyl-4H-cyclopenta[2,1-b:3,4-b']dithiophen-2-yl)isoquinoline (5)


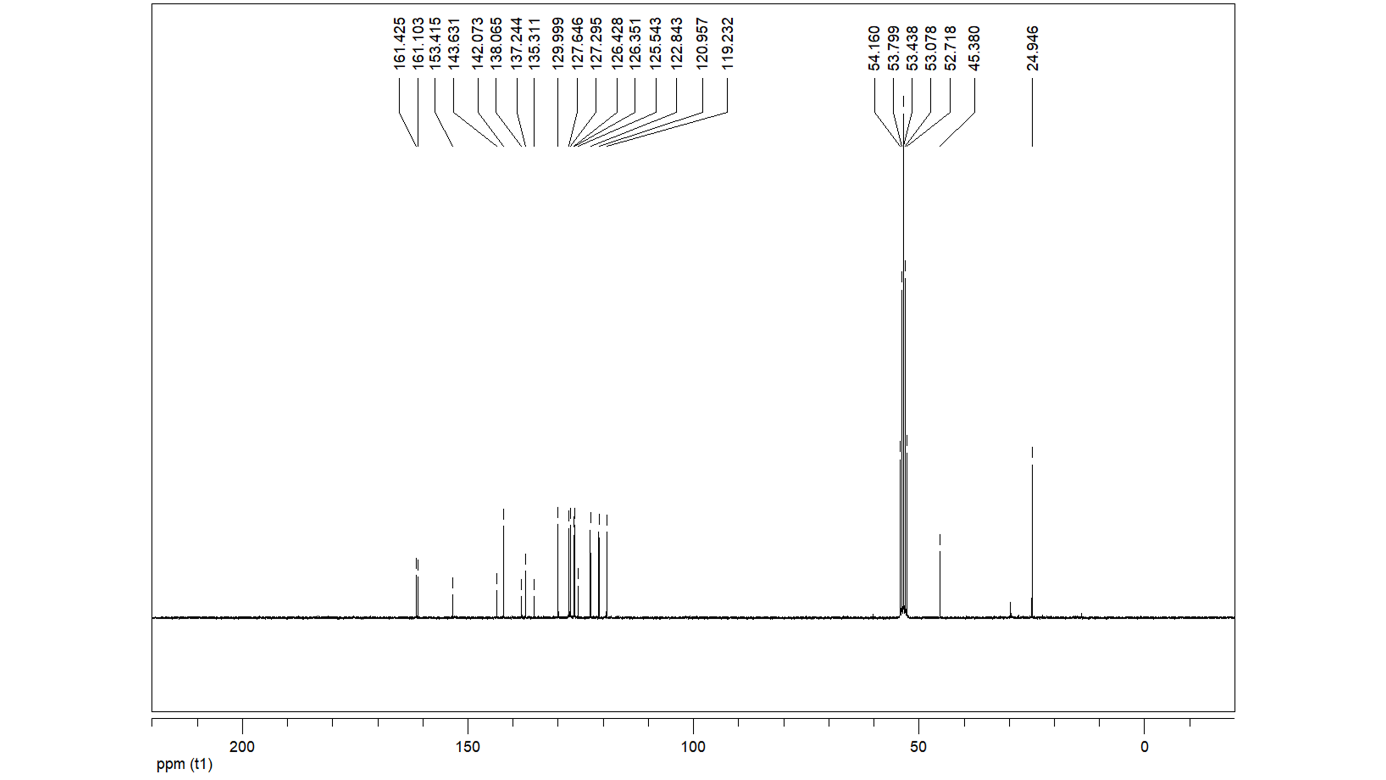


**Figure S6.** ^13^C-NMR data of 1-(4,4-dimethyl-4H-cyclopenta[2,1-b:3,4-b']dithiophen-2-yl)isoquinoline (5)


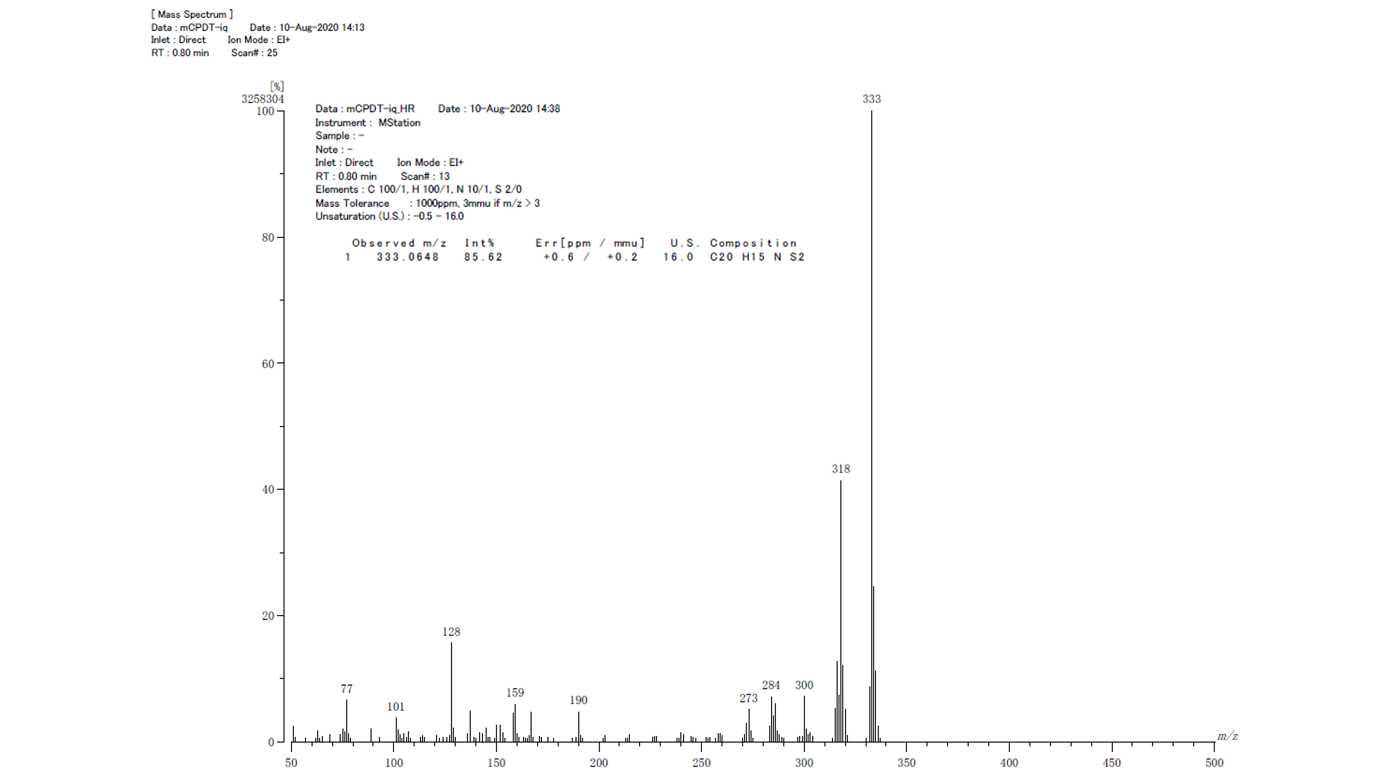


**Figure S7.** FAB mass data of 1-(4,4-dimethyl-4H-cyclopenta[2,1-b:3,4-b']dithiophen-2-yl)isoquinoline (5)


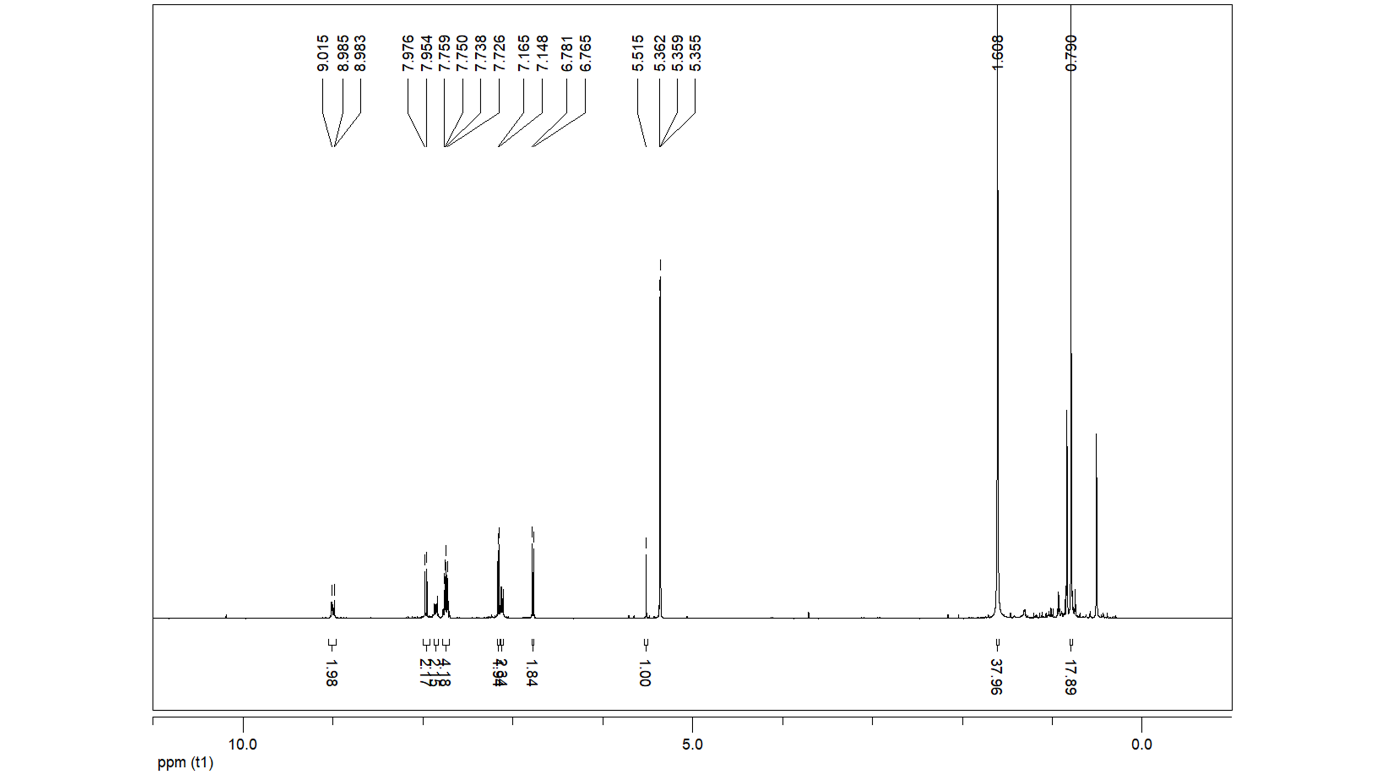


**Figure S8.** ^1^H-NMR data of Ir(mCPDTiq)_2_tmd


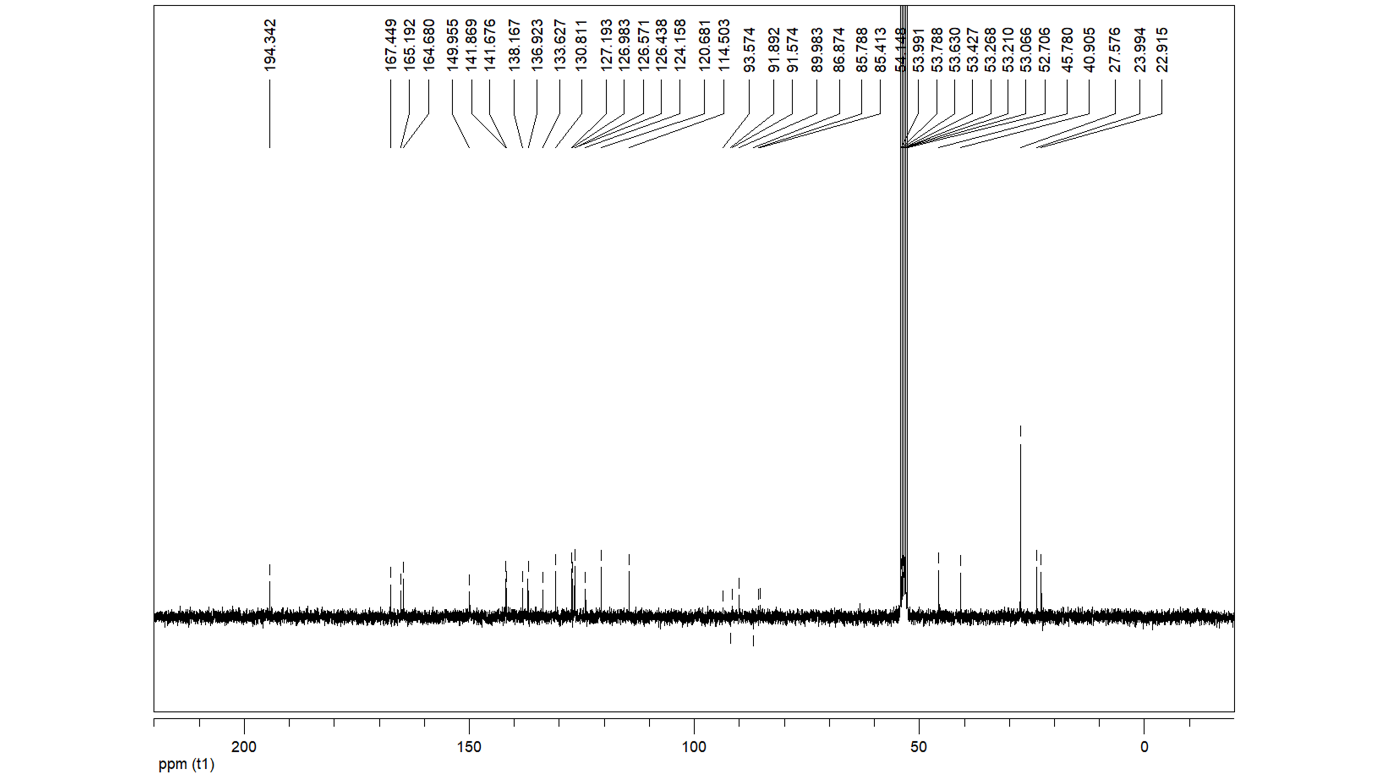


**Figure S9.** ^13^C-NMR data of Ir(mCPDTiq)_2_tmd

**
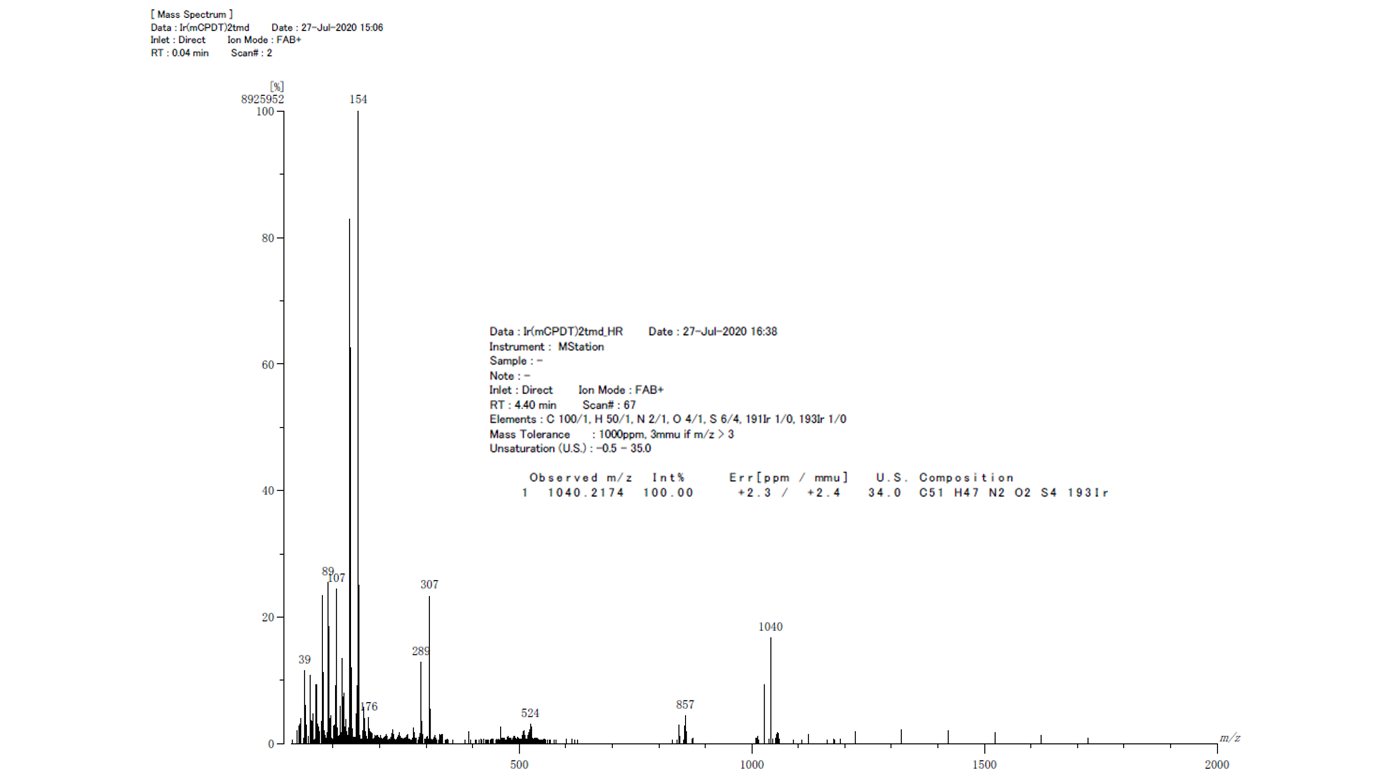
**

**Figure S10.** FAB mass data of Ir(mCPDTiq)_2_tmd


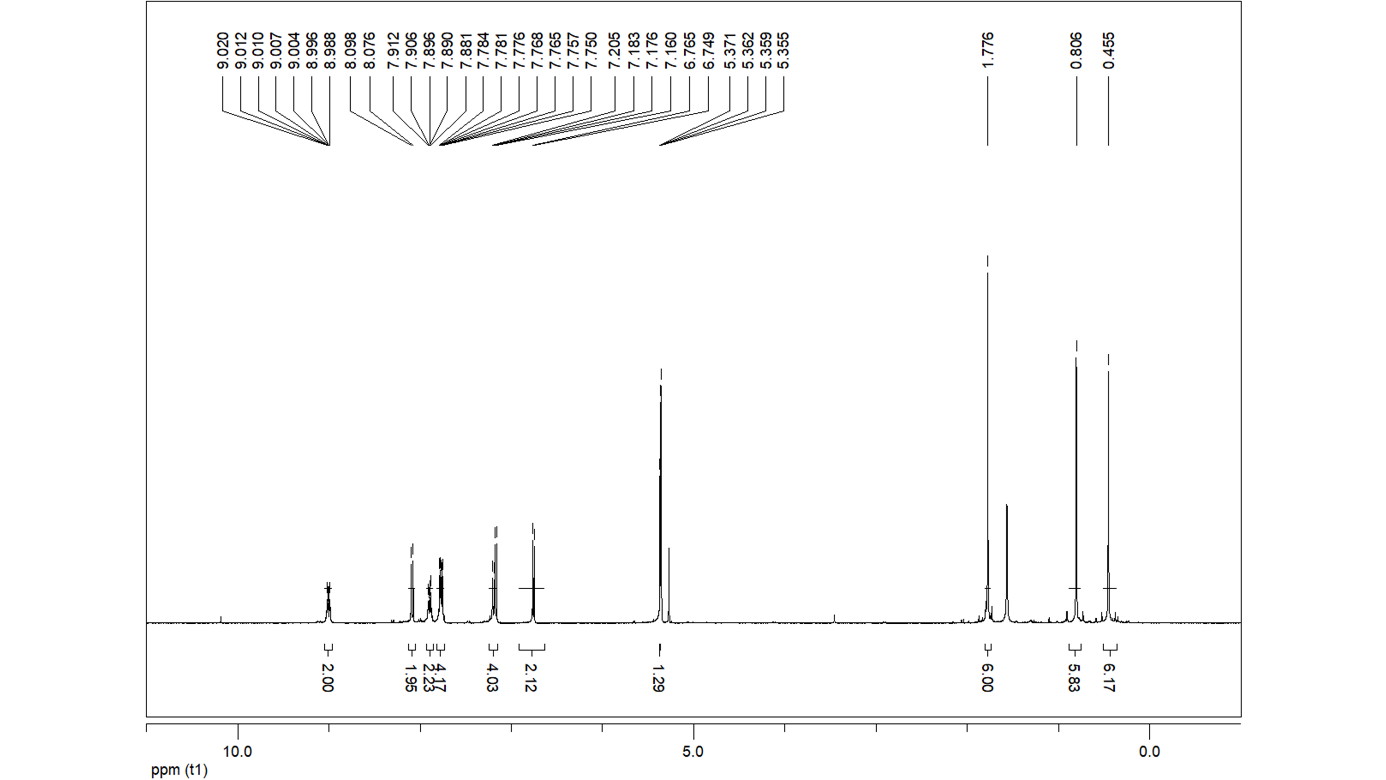


**Figure S11.** ^1^H-NMR data of Ir(mCPDTiq)_2_acac


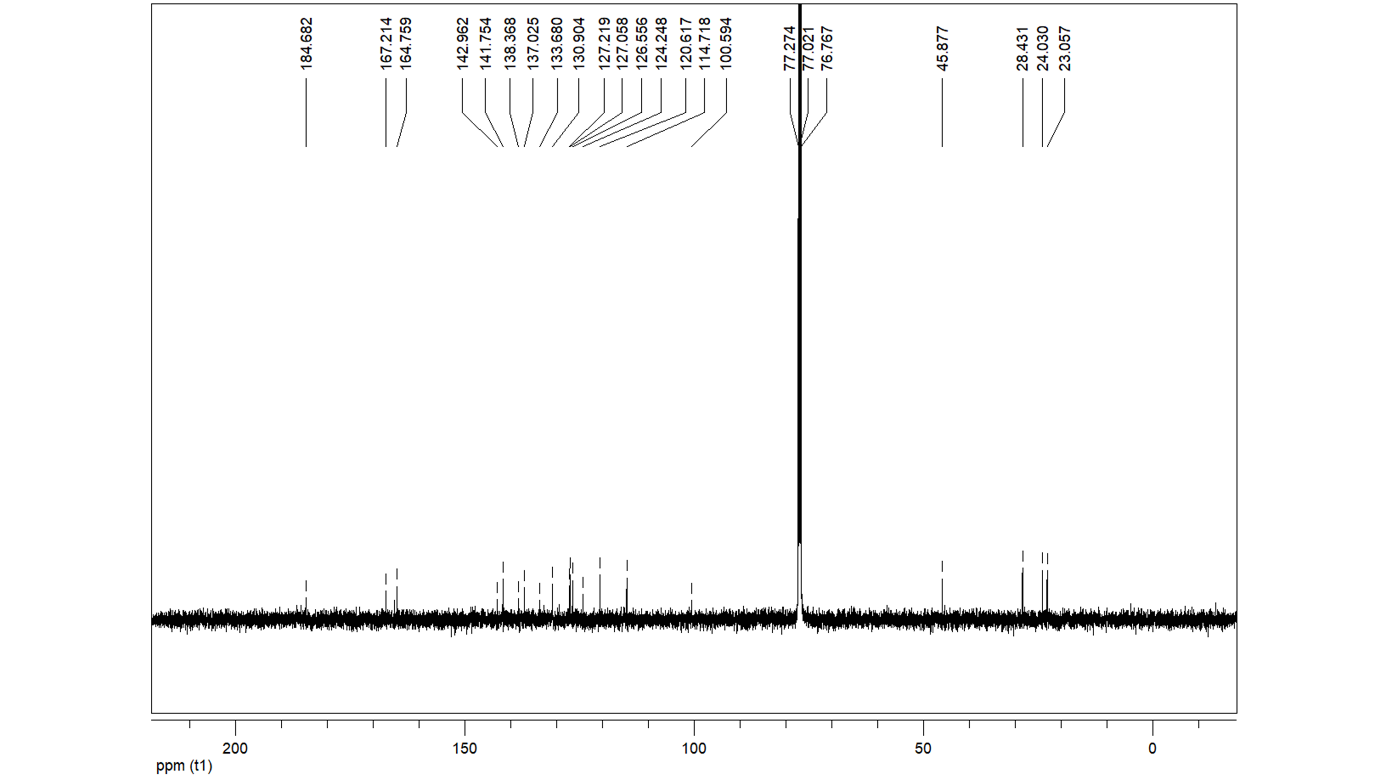


**Figure S12.** ^13^C-NMR data of Ir(mCPDTiq)_2_acac


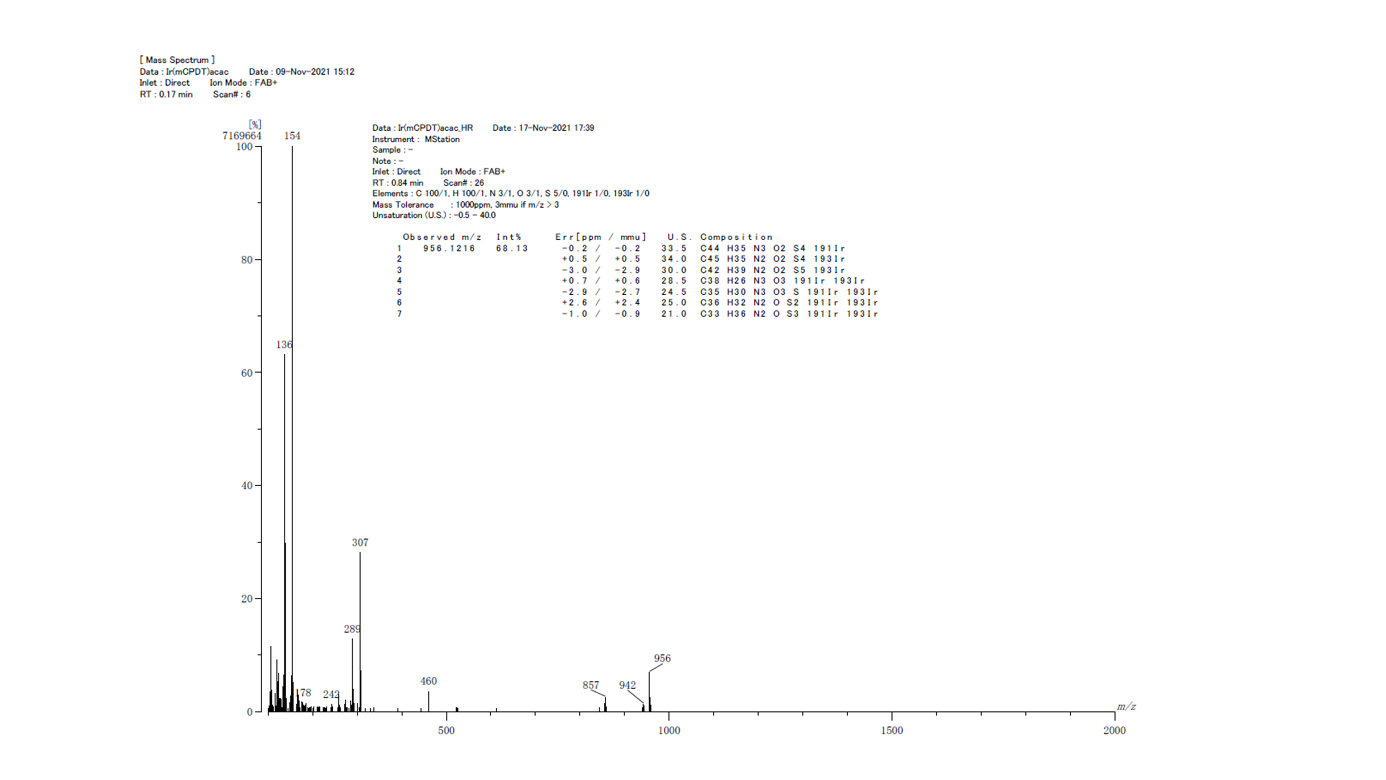


**Figure S13.** FAB mass data of Ir(mCPDTiq)_2_acac





**Figure S14.** TGA thermogram of Ir(mCPDTiq)_2_tmd





**Figure S15~~.~~** TGA thermogram of Ir(mCPDTiq)_2_acac





**Figure S16.** DSC thermogram of Ir(mCPDTiq)_2_tmd





**Figure S17.** DSC thermogram of Ir(mCPDTiq)_2_acac


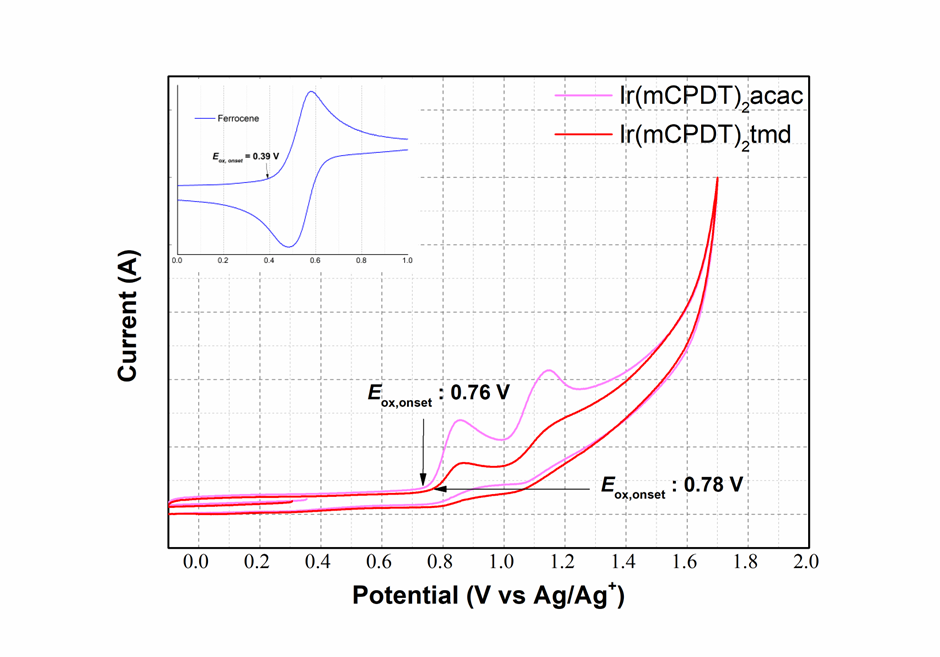


E_HOMO_ = - (4.84 – E_FOC_ + E_onset_ (OX)) (eV)

E_FOC_ is the potential of FOC/FOC + vs. Ag/AgCl (= 0.39 eV)

Ir(mCPDT)_2_tmd ------------------------------------------------------

E_HOMO_ = – (4.84 – 0.39 + 0.78) = – 5.23 eV

E_LUMO_ = – (E_HOMO_ – E_g_) = – (5.30 – 2.08) = – 3.15 eV

Ir(mCPDT)_2_acac -----------------------------------------------------

E_HOMO_ = – (4.84 – 0.39 + 0.76) = – 5.21 eV

E_LUMO_ = – (E_HOMO_ – E_g_) = – (5.30 – 2.08) = – 3.13 eV

**Figure S18.** Cyclic voltammetry analysis of the iridium complexes


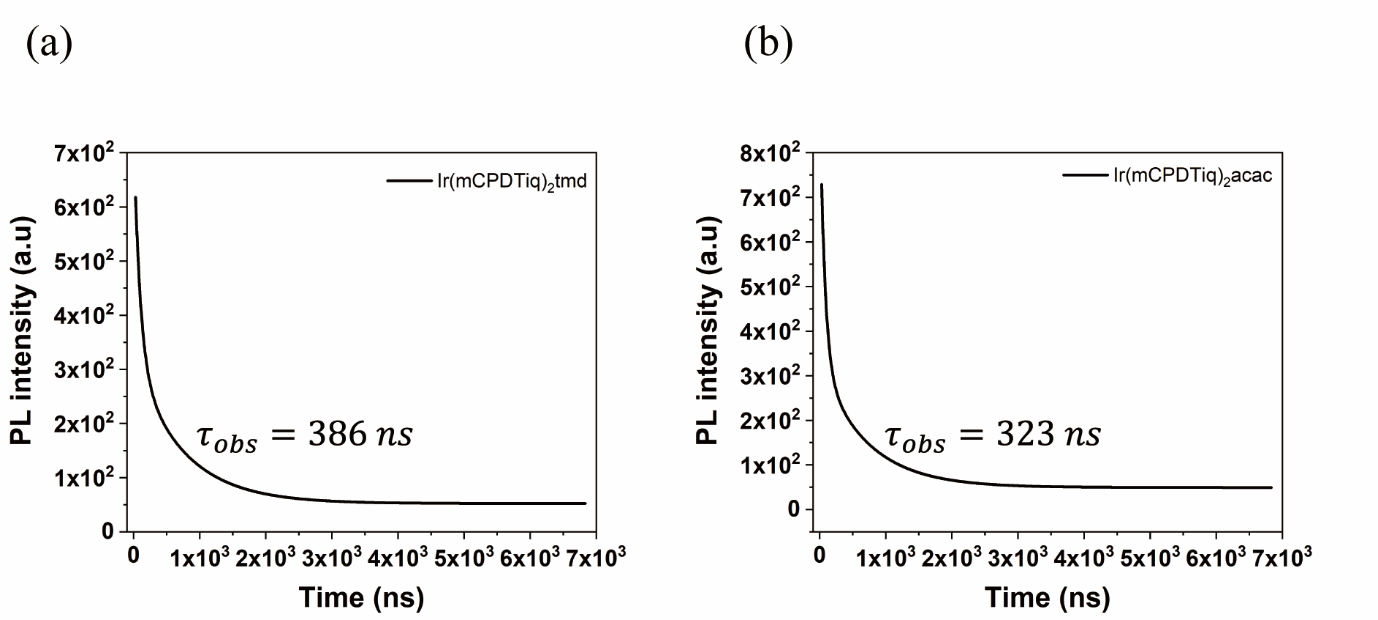


**Figure S19.** Transient PL decay of complex **1** and **2** measured at a room temperature (298 K).





**Figure S20.** PL spectra of host (Bebq_2_) in vacuum-deposited thin-film (30 nm on glass). Excitation wavelength was set to 275 nm.





**Figure S21.** PL of thin-film (30 nm) on boron doped Si wafer depending on doping concentration. Excitation wavelength was set to 320 nm.





**Figure S22.** Angular dependence graph of NIR OLEDs. All devices exhibit a Lambertian distribution.


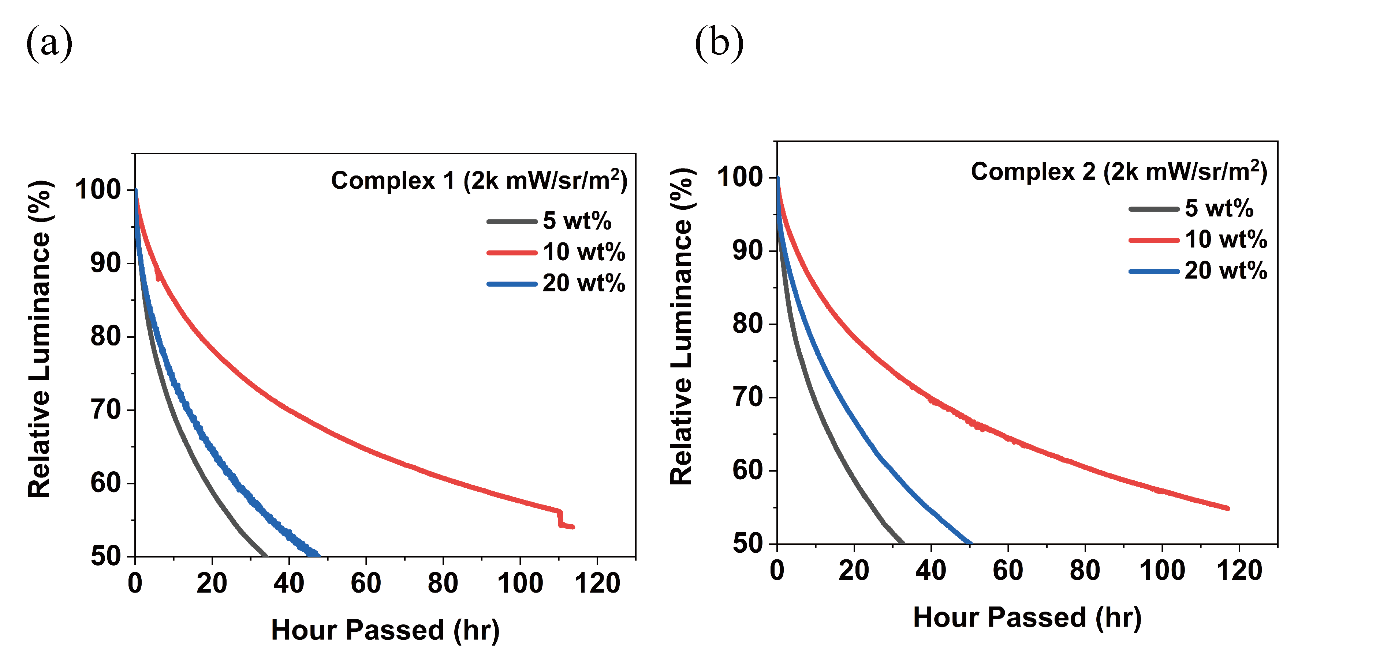


**Figure S23.** Graph of relative luminance over time of NIR OLEDs depending on the doping concentration at a radiance of 2k mW/sr/m^2^.

**Table S1.** Values of the wavelength and PLQY of the Ir(III) complex based previous research.

| Ref | Wavelength  (nm) | PLQY  (%) |
| --- | --- | --- |
| This work | 760 | 3.2 (complex 1, film)  3.0 (complex 2, film) |
| 25 | 748 | 30 (film) |
| 26 | 760 | 14.7 (solution) / 17.3 (film) |
| 27 | 760 | 8 (solution) |
| 28 | 775 | 2.9 (solution) / 4.1 (film) |
| 29 | 777 | 1.5 (solution) / 3.6 (film) |
| 30 | 847 | 1.5 (solution) / 1.9 (film) |

**Table S2.** Numerical data reported in this and previous studies (Unit of radiance was unified as mW/sr/m^2^, and if the exact value was not indicated in the previous studies, the value was confirmed through a graph)

| Ref | R_max_  (mW/sr/m^2^) | V_max_  (V) | RC (mW/sr/m^2^/V) |
| --- | --- | --- | --- |
| This work | 5060 | 7 | 723 |
| 25 | 1000 | 14 | 71 |
| 26 | 13300 | 20 | 665 |
| 27 | 11518 | 25 | 461 |
| 28 | 0.018 | 20 | 0.009 |
| 29 | 5730 | 15 | 382 |
| 30 | 297 | 19 | 16 |
